# Optogenetic control of the Dab1 signaling pathway

**DOI:** 10.1038/srep43760

**Published:** 2017-03-08

**Authors:** Liang Wang, Jonathan A. Cooper

**Affiliations:** 1 Division of Basic Sciences, Fred Hutchinson Cancer Research Center, 1100 Fairview Ave N, Seattle, Washington 98109, USA

## Abstract

The Reelin-Dab1 signaling pathway regulates development of the mammalian brain, including neuron migrations in various brain regions, as well as learning and memory in adults. Extracellular Reelin binds to cell surface receptors and activates phosphorylation of the intracellular Dab1 protein. Dab1 is required for most effects of Reelin, but Dab1-independent pathways may contribute. Here we developed a single-component, photoactivatable Dab1 (opto-Dab1) by using the blue light-sensitive dimerization/oligomerization property of *A. thaliana* Cryptochrome 2 (Cry2). Opto-Dab1 can activate downstream signals rapidly, locally, and reversibly upon blue light illumination. The high spatiotemporal resolution of the opto-Dab1 probe also allows us to control membrane protrusion, retraction and ruffling by local illumination in both COS7 cells and in primary neurons. This shows that Dab1 activation is sufficient to orient cell movement in the absence of other signals. Opto-Dab1 may be useful to study the biological functions of the Reelin-Dab1 signaling pathway both *in vitro* and *in vivo*.

The Reelin signaling pathway regulates neuron migrations that are important for the characteristic layered architecture of the mammalian neocortex, cerebellum and hippocampus, and also regulates synaptic plasticity in the adult brain^1^,^2^. Reelin is a large extracellular protein secreted by the Cajal-Retzius cells in the marginal zone of the neocortex during development and by interneurons after birth. It can bind to the single-pass transmembrane receptors very-low density lipoprotein receptor (VLDLR) and ApoE receptor 2 (ApoER2) on the neuronal surface and induces the clustering of these receptors. Dab1, an adaptor protein in the cytosol, binds to the cytoplasmic tails of both receptors and is phosphorylated by the Src family kinases (SFKs) Src or Fyn following Reelin stimulation^3^,^4^. The activated Dab1 (pY Dab1) then recruits the p85 subunit of phosphatidylinositol 3-kinase (PI3K) and the adaptor proteins Crk and CrkL, which are complexed with C3G, a Rap1 guanine nucleotide exchange factor (GEF)^5^,^6^. These events activate PI3K and Rap1, which together can activate multiple small GTPases, including Rac1, Cdc42 and RalA. Reelin signals converge to induce remodeling of the actin cytoskeleton and direct the traffic of intracellular vesicles^7^,^8^,^9^. Experiments using dominant negative mutants or RNA interference *in vivo* suggest that these events are required at least twice during migration of cortical projection neurons^8^,^10^,^11^,^12^,^13^. The first requirement is in the intermediate zone (IZ), where Reelin induces the polarization of multi-polar neurons^8^. The second stage is in the marginal zone (MZ), where Reelin stimulates extension and attachment of the leading process of the neuron and induces somal translocation and maturation of the dendritic tree^10^,^11^,^14^. Both these stages involve polarized outgrowth of a leading process, suggesting that Reelin, acting alone or together with other signals, is able to induce and perhaps orient membrane and cytoskeletal protrusion.

It is unclear whether Reelin acts locally, perhaps as a direction signal to orient neuron polarization, or globally, to sensitize neurons to other direction signals. Tools to activate Reelin signaling locally, in one part of a neuron, may be useful to distinguish these possibilities. Förster and colleagues localized Reelin by making stripes of Reelin on a membrane, and performed a stripe-choice assay with radial glial cells from the dentate gyrus^15^. This assay showed that glial cells but not other cells migrated along Reelin stripes. However, the assay has not been widely adopted. Reelin could also be applied locally by a mechanical method, for example by use of microfluidics^16^, but this has not, to our knowledge, been attempted. Strasser and colleagues made a Dab1 fusion protein that can be dimerized inside the cell by a chemical, AP20187^17^. In principle, AP20187 could also be applied locally. However, diffusion of locally applied Reelin or AP20187 could limit the spatial resolution of the assay. Recently, there has been significant improvement in the field of optogenetics, and light-sensitive probes have been developed to activate signaling pathways *in vitro* or *in vivo*^18^,^19^. We tested whether optogenetic induction of Dab1 oligomerization, mimicking its clustering by Reelin, would cause local activation of Dab1 signaling. Optically-induced clustering was achieved using the photolyase homology region (PHR) domain of *A. thaliana* Cryptochrome 2 (Cry2) protein, which forms dimers and higher-order oligomers with sub-second kinetics when illuminated with blue light (~400 nm−500 nm), and then dissociates in minutes in the dark^20^,^21^,^22^. In this study we fused Dab1 with Cry2olig (a more robust version of Cry2PHR^22^) and exploited the light-induced signaling properties of this photoactivatable Dab1. We showed that Dab1 oligomerization leads to phosphorylation of Dab1, activates downstream signals, and induces actin cytoskeleton remodeling and membrane protrusion in a variety of cell types, including neurons. The results suggest that Reelin may be a directional signal for neurons *in vivo*.

## Results

### Light-induced membrane targeting and phosphorylation of opto-Dab1

**Figure 1:**
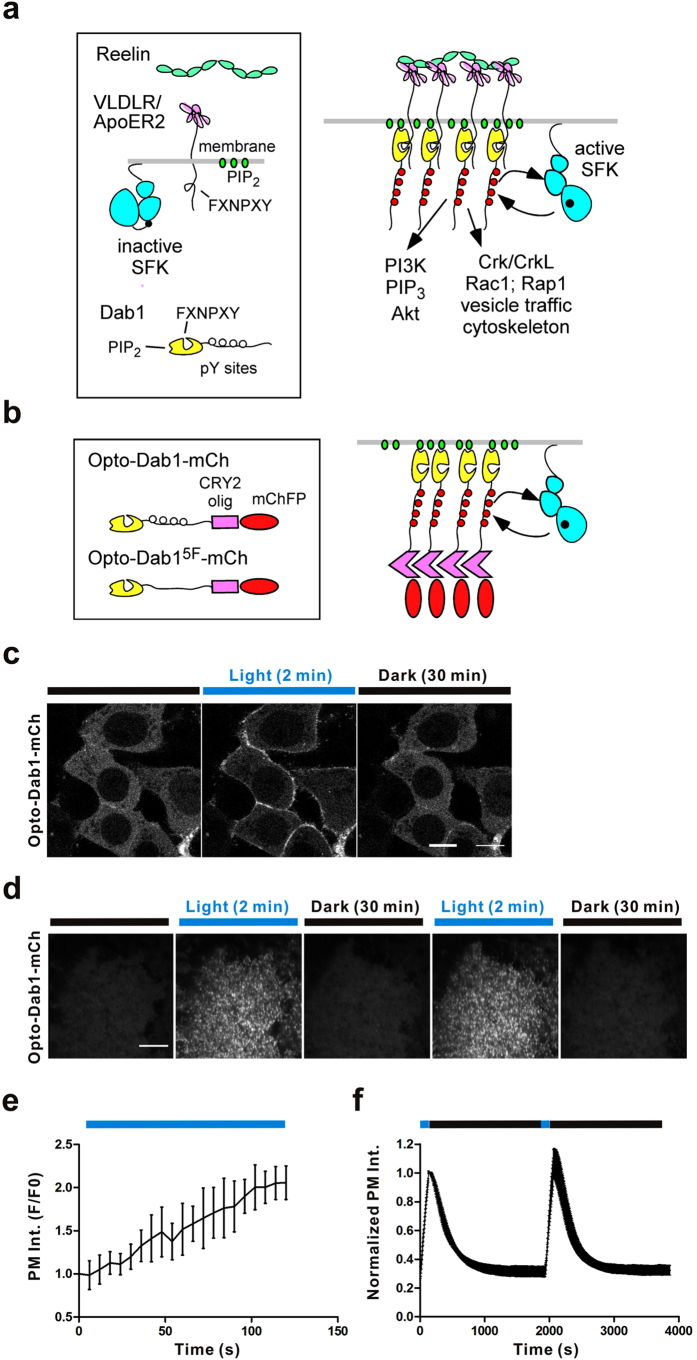
Plasma membrane recruitment and phosphorylation of opto-Dab1 induced by blue light in cells. (**a**) Schematic of the Reelin signaling pathway activation. Reelin binds to VLDLR/ApoER2 receptors and induces the phosphorylation of Dab1, which recruits and activates PI3K and Crk proteins. (**b**) Schematic of the Dab1-Cry2olig-FP fusion (designated as opto-Dab1-FP) and the 488 nm light-induced phosphorylation of opto-Dab1-FP and activation of downstream effectors. (**c**) Confocal images (6 s/frame) showing the translocation of opto-Dab1-mCh (561 nm) upon 488 nm laser line irradiation and fluorescence recovery without 488 nm laser illumination in HEK293 cells. (**d**) TIRF images showing the membrane recruitment and puncta formation of opto-Dab1-mCh (561 nm) upon 488 nm laser line irradiation and fluorescence recovery without 488 nm laser illumination in COS7 cells. (**e–f**) Quantification of the data in (**c**) (n = 7 cells) and (**d**) (n = 4 cells), respectively. Error bars show the standard deviation (S.D.). Scale bars: 10 μm.

During central nervous system development, extracellular Reelin binds to the VLDLR and ApoER2 receptors on the neuron surface, which then activate SFKs and phosphorylation of Dab1. pY Dab1 then acts as a key adaptor to recruit and activate immediate downstream effectors near the membrane (Fig. 1a). To achieve optogenetic control of the Reelin signaling pathway, we constructed opto-Dab1, containing the mouse Dab1 protein, the blue light-sensitive portion (PHR domain) of Cry2olig and a fluorescent protein (FP, such as mCherry, GFP or EYFP) (Fig. 1b). To examine the properties of opto-Dab1-FP, we first expressed opto-Dab1-mCh in HEK293 cells and examined its location by confocal microscopy. Before 488 nm laser illumination, opto-Dab1-mCh was cytosolic. However, following 488 nm laser illumination (line scan at 0.2% power every 6 s), opto-Dab1-mCh relocalized to the plasma membrane in all cells examined (Fig. 1c). Relocalization was rapid (t_1/2_ ~ 50 s, Fig. 1e). Membrane recruitment was reversible since opto-Dab1-mCh returned to the cytosol in the dark (t_1/2_ ~ 10 min, Fig. 1c,f). We reasoned that membrane translocation may result from opto-Dab1 clustering, which would increase the local concentration of the Dab1 PTB domain and the binding avidity for phosphatidylinositol-4,5-bisphosphate in the plasma membrane^17^,^23^,^24^. To test whether microscopic clusters formed, we used total internal reflectance (TIRF) microscopy to observe the ventral surface of the cells. Illumination at 488 nm induced opto-Dab1-mCh translocation and the formation of puncta, suggesting membrane targeting and cluster formation (Fig. 1d,f). These processes were reversible when 488 nm laser illumination was halted (Fig. 1d,f).

We tested whether clustering was sufficient to induce opto-Dab1 phosphorylation in the absence of Reelin signals. We expressed opto-Dab1-mCh and a phosphorylation-defective control opto-Dab1^5F^-mCh, which lacks the known sites of Reelin-induced phosphorylation^25^. After 16 hr starvation in FBS-free DMEM, the cells were illuminated with repeated light flashes from an array of blue LEDs. Western blot analysis showed an obvious increase in phosphorylation of opto-Dab1-mCh. Phosphorylation of opto-Dab1^5F^-mCh was not increased (Supplementary Fig. S1). This indicates that Reelin and optogenetic activation induce the phosphorylation of the same tyrosine residues in Dab1, suggesting that opto-Dab1-FP bypasses Reelin for Dab1 activation.

### Activation of opto-Dab1 induces PI3K activation and membrane recruitment of Crk protein

**Figure 2:**
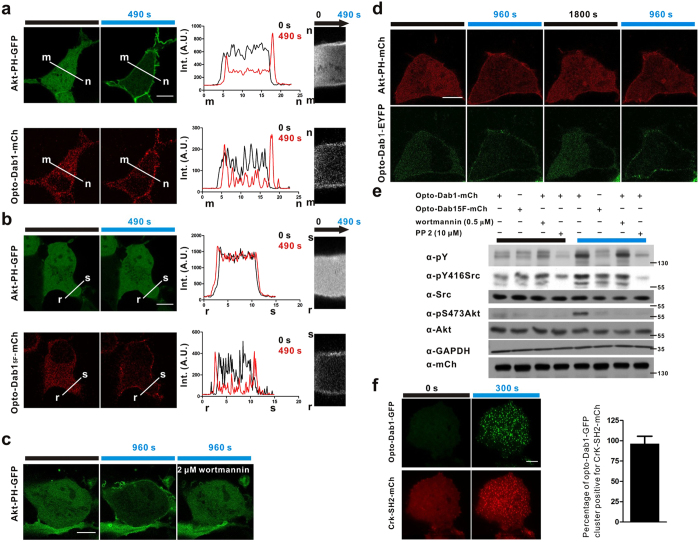
PI3K activation and Crk recruitment induced by global irradiation of opto-Dab1-FP in COS7 cells. (**a**–**b**) Serum-starved COS7 cells co-expressing Akt-PH-GFP and opto-Dab1-mCh (**a**) or opto-Dab1^5F^-mCh (**b**) were subjected to global illumination at 488 nm (10 s/frame). The images and the intensity distribution along the white lines at zero time point and 490 s time point are shown on the left. Kymographs along the white lines m-n and r-s are shown on the right. (**c**) Serum-starved COS7 cells co-expressing opto-Dab1-mCh and Akt-PH-GFP were irradiated at 488 nm for 32 min (10 s/frame). 2 μM wortmannin was added immediately after the 16 min time point. Confocal images of Akt-PH-GFP at 0 min, 16 min and 32 min are shown. (**d**) Reversibility of light-induced PI3K activation. Serum-starved COS7 cells co-expressing opto-Dab1-EYFP and Akt-PH-mCh were irradiated globally at 488 nm for 16 min (10 s/frame), then relaxed without blue light irradiation for 30 min, and then irradiated again for 16 min. Confocal images of red (561 nm) and green (488 nm) signals are shown at the indicated time points. (**e**) Serum-starved COS7 cells expressing opto-Dab1-mCh or opto-Dab1^5F^-Cry2olig-mCh were treated with 0.5 μM wortmannin or 10 μM PP2 or untreated, and then subjected to blue light irradiation with a LED array for 20 mins (on/off, 0.05 s/10 s) or left in the dark. Cells were lysed, analyzed by Western blotting, and probed for phosphotyrosine, active Akt, GAPDH (loading control) and mCherry. (**f**) Serum-starved COS7 cells co-expressing opto-Dab1-GFP and Crk-SH2-mCh were irradiated globally with 488 nm laser line under TIRFM and the TIRF images at time point 0 s and 300 s are shown. The quantification of co-localization was shown on the right (n = 28 cells). Scale bars: 10 μm.

Based on the above results and reported studies^6^, we hypothesized that membrane-binding and phosphorylation of opto-Dab1-FP would recruit the PI3K subunit p85 to the plasma membrane and activate the p110 catalytic subunit, which produces phosphatidylinositol (3,4,5) trisphosphate (PIP_3_) and then recruits and activates Akt kinase. To test this, we expressed opto-Dab1-mCh or opto-Dab1^5F^-mCh together with Akt-PH-GFP (a probe for PI(3,4,5)P_3_ production) in COS7 cells. After 6–8 hours starvation in FBS-free DMEM, opto-Dab1-mCh, opto-Dab1^5F^-mCh and Akt-PH-GFP were all cytosolic. Within ~60 s of 488 nm illumination of cells expressing opto-Dab1-mCh and Akt-PH-GFP, both proteins started to translocate to the membrane (Fig. 2a). However, illumination of cells expressing opto-Dab1^5F^-mCh and Akt-PH-GFP induced membrane translocation of opto-Dab1^5F^-mCh but not Akt-PH-GFP (Fig. 2b). 488 nm illumination did not induce membrane translocation of Akt-PH-mCh in cells expressing Akt-PH-mCh only (Supplementary Fig. S2a). This suggests that Akt-PH membrane translocation requires clustering and tyrosine phosphorylation of opto-Dab1.

To test whether Akt-PH translocation requires PI3K activity, we added 2 μM wortmannin to inhibit PI3K activity during the 488 nm laser illumination. Akt-PH-GFP did not translocate to the membrane under these conditions, suggesting that opto-Dab1 activation and tyrosine phosphorylation stimulates PI3K to recruit Akt-PH-GFP to the membrane (Fig. 2c).

Since the translocation of opto-Dab1 is reversible, we hypothesized that the activation of PI3K may also be reversible. Indeed, Akt-PH-mCh was released from the membrane in the dark (Fig. 2d). Reversible relocalization of Akt-PH-mCh was confirmed by TIRF imaging (Supplementary Fig. S2b). This suggests that membrane PIP_3_ is rapidly hydrolyzed when PI3K is no longer active. Both Dab1-Cry2olig and Akt-PH-mCh could be activated repeatedly by cycles of light and dark (Fig. 2d).

We tested whether endogenous Src and Akt were activated by opto-Dab1 using Western blotting. Indeed, Src family kinase phosphorylation at Tyr 419 and Akt phosphorylation at Ser 473 were induced by blue light illumination of COS7 cells expressing opto-Dab1 (Fig. 2e). Akt phosphorylation was inhibited by wortmannin. We tested whether activation of Akt by opto-Dab1 requires Src family kinases using the Src family inhibitor PP2. PP2 inhibited phosphorylation of opto-Dab1, Src and Akt. The results suggest that clustered, but not free, opto-Dab1 activates Src family kinases, which then activate PI3K and Akt (Fig. 2e).

Since pY Dab1 can recruit Crk proteins and activate Rap1^5^, we test whether activated opto-Dab1-FP can recruit Crk. To this end we made a probe containing the SH2 domain from Crk and mCherry (Crk-SH2-mCh)^26^. We co-expressed this probe and opto-Dab1-GFP in COS7 cells. TIRF imaging showed that 488 nm illumination induced co-clustering of opto-Dab1 with the Crk SH2 domain (Fig. 2f). Crk-SH2-mCh was not recruited to the membrane or clustered when opto-Dab1^5F^-GFP was activated (Supplementary Fig. S2c). These data suggested that each opto-Dab1-GFP cluster is a functional unit for signal transduction.

### Local Dab1 activation leads to local signal activation and membrane protrusion

**Figure 3:**
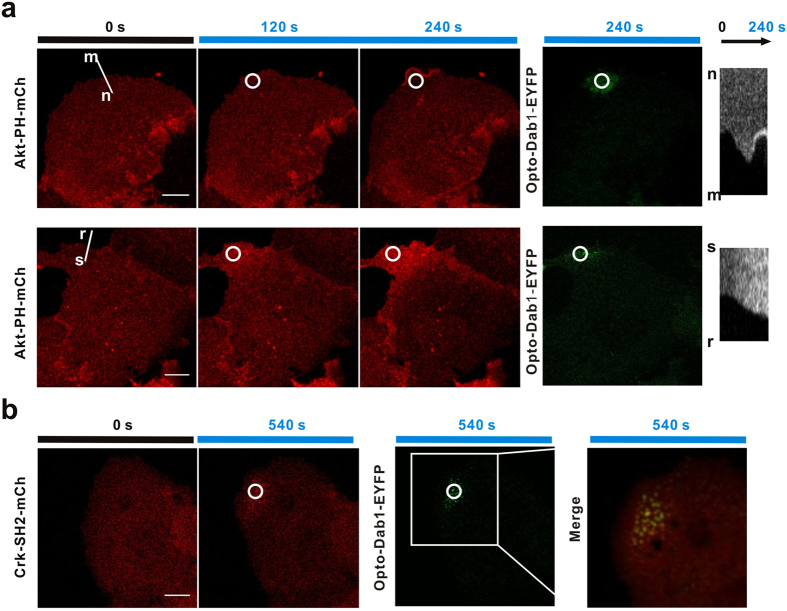
Local PI3K activation and Crk-SH2 translocation induced by local irradiation of opto-Dab1-FP in COS7 cells. (**a**) COS7 cells co-expressing opto-Dab1-EYFP and Akt-PH-mCh were serum starved for 16 h, then a ~6.6 μm diameter spot (indicated by a white circle) was repeatedly irradiated with a 488 nm laser, with images recorded at 561 nm. Images show Akt-PH-mCh at 0, 120 s and 240 s. The entire cell was then imaged at 488 nm. The upper and lower rows show results from two different cells. Right: kymographs of Akt-PH-mCh along the white lines m-n and r-s. (**b**) Serum-starved COS7 cells co-expressing opto-Dab1-EYFP and Crk-SH2-mCh were irradiated locally (white circle), then imaged at 561 nm. Images show Crk-SH2-mCh at 0 s and 540 s, and the entire cell imaged at 488 nm at 540 s. The merged image shows co-localization of opto-Dab1-EYFP and Crk-SH2-mCh at 540 s. Frame rate: 6 s/frame. Scale bars: 10 μm.

One of the most important advantages of optogenetic probes is their high spatial-temporal resolution. To test whether our opto-Dab1-FP probe can be activated with a high spatial resolution, we co-expressed opto-Dab1-EYFP and Akt-PH-mCh in COS7 cells and activated a small region (diameter ~6.6 μm) near the cell edge with a 488 nm laser. Opto-Dab1 was recruited to the immediate vicinity and Akt-PH-mCh was recruited to the illuminated region and up to ~10 μm distant (Fig. 3a). This suggests that PIP_3_ may diffuse a short distance from the site of opto-Dab1 activity and recruit Akt-PH to the membrane over a larger region. Akt-PH-mCh intensity often decreased in other parts of the activated cell, suggesting that the probe may be limiting. Local activation of opto-Dab1-EYFP also recruited Crk-SH2-mCh, forming puncta that co-localized with opto-Dab1 puncta (Fig. 3b). Together, these results demonstrated that local activation of opto-Dab1-FP leads to local signal activation, with different spatial ranges for different downstream signals.

**Figure 4:**
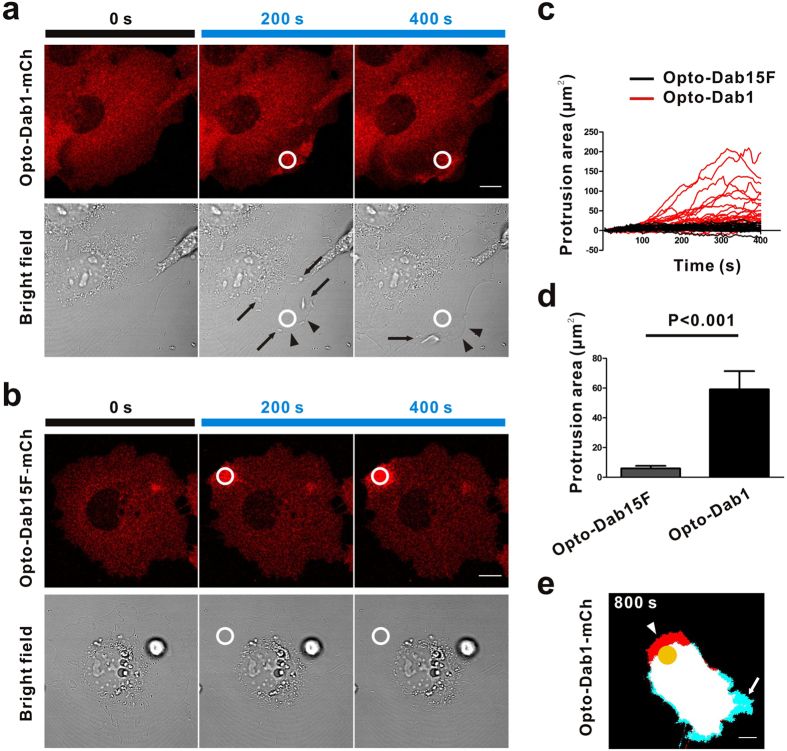
Cell mobility changes induced by local irradiation of opto-Dab1-mCh in COS7 cells. (**a–b**) COS7 expressing opto-Dab1-mCh (**a**) or opto-Dab1^5F^-mCh (**b**) were serum starved for 16 h then a ~6.6 μm diameter spot (indicated by a white circle) was repeatedly irradiated with a 488 nm laser and images recorded at 561 nm and with bright field optics. Ruffles and protrusions are indicated by black arrows and black arrowhead, respectively. (**c**) Graphs show protrusion area (area change around the activation spot) against time. Red lines (n = 28 cells) and black lines (n = 21 cells) indicate cells expressing opto-Dab1-mCh and opto-Dab1^5F^-mCh, respectively. (**d**) Quantification of membrane area increase after 400 s of repeated illumination of opto-Dab1^5F^-mCh and opto-Dab1-mCh cells. (**e**) Protrusion/retraction map of a COS7 cell expressing opto-Dab1-mCh by local activation for 800 s. Protrusion (red, arrowhead) was induced at the activation site and retraction (cyan, arrow) at the opposite side. Frame rate: 5 s/frame. Scale bars: 10 μm.

PI3K activation and Crk protein membrane translocation can activate the Rap1, Cdc42 and Rac1 GTPases, which lead to membrane ruffling, lamellipodia and/or filopodia formation^6^,^7^,^27^,^28^,^29^. We hypothesized that local activation of opto-Dab1-FP may induce cell motility. We expressed opto-Dab1-mCh in COS7 cells and stimulated near one edge of the cell. Repeated local blue light illumination induced edge/dorsal membrane ruffling and cell edge protrusion close to the irradiated area (Fig. 4a). Ruffling started at the cell edge closest to the activation point and then spread locally (Movie S1). In some COS7 cells, we found that local activation induced retraction at the opposite side (Fig. 4e, Movie S2). Moving the illumination spot to a different location induced protrusions at the new spot (Supplementary Fig. S2d). As a control, local activation of opto-Dab1^5F^-mCh did not induce membrane ruffling or cell edge protrusion (Fig. 4b–d, Movie S3). Together these results demonstrated that opto-Dab1-FP can induce directional membrane protrusion. Also persistent local activation of opto-Dab1 in NIH3T3 cells led to prolonged movement (Movie S4), which indicates that opto-Dab1 is able to induce cell polarity.

### Signaling and membrane protrusion induced by opto-Dab1 in primary cortical neurons

**Figure 5:**
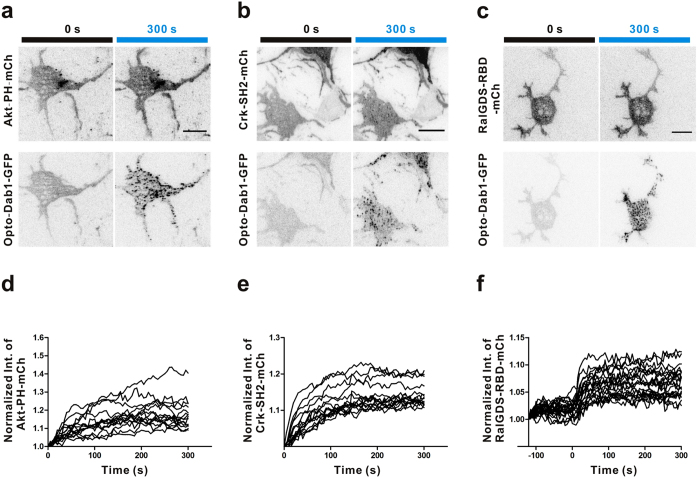
Activation of downstream effectors in primary cortical neurons after global illumination under TIRF. (**a**–**c**) Primary cortical neurons were electroporated with opto-Dab1-GFP and either Akt-PH-mCh (**a**), Crk-SH2-mCh (**b**), or RalGDS-RBD-mCh (**c**), and incubated for 2 days before starving for 6 h in the absence of B27. Cells were irradiated at 488 nm and imaged at 488 and 561 nm under TIRF optics. Images at 0 s and 300 s are shown. (**d**–**f**) Quantification of intensity changes in (**a**–**c**), and n = 14 cells, 15 cells, 21 cells, respectively. Scale bars: 10 μm.

**Figure 6:**
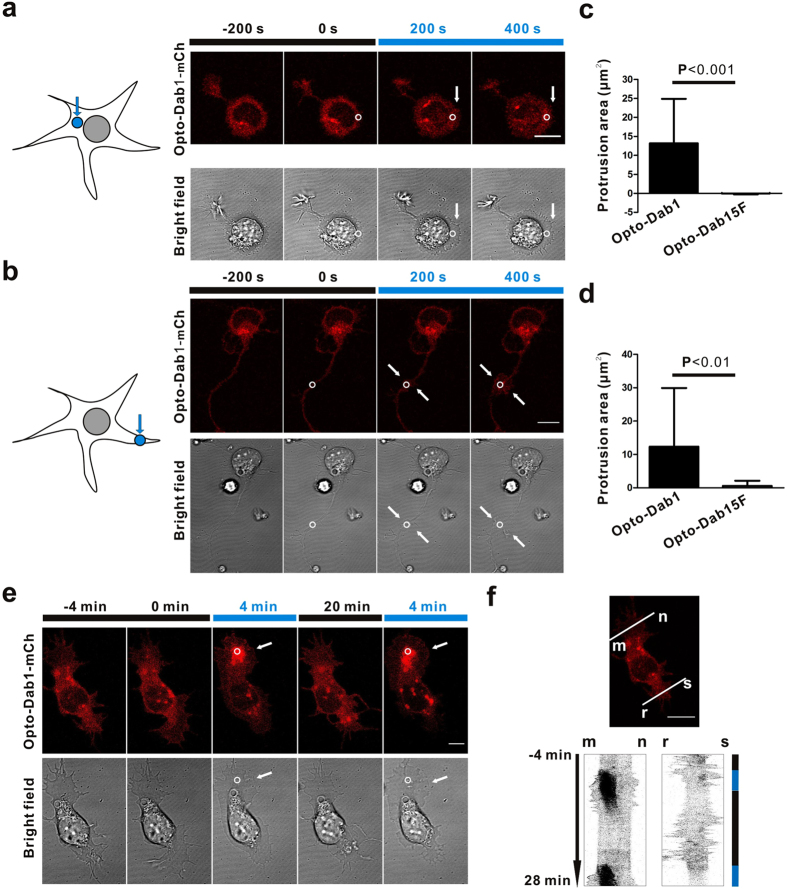
Membrane protrusion induced by local irradiation of opto-Dab1-mCh in cortical neurons. (**a**–**b**) Membrane protrusion induced by blue light illumination of neuron cell body or neurites. Neurons were nucleofected with opto-Dab1-mCh, incubated for one day, and starved in B27-free medium for 6 h. They were then subjected to repeated local activation by a 488 nm laser line spot indicated by a white circle (with a diameter ~1.7 μm). The spot was positioned at the cell body (**a**) or at the neurite (**b**). Confocal images (561 nm and bright field optics) were taken 200 s before and 0, 200 and 400 s after activation. White arrows indicate the protrusion area. (**c**,**d**) Quantification of protrusion area at the end of the activation process for illumination at the cell body (c, n = 31 cells for opto-Dab1-mCh and n = 21 cells for opto-Dab1-^5F^-mCh) or at a neurite (d, n = 30 cells for opto-Dab1-mCh and n = 22 cells for opto-Dab1-^5F^-mCh). (**e**) Reversible protrusion induced by local activation opto-Dab1-mCh in a neuron. A transfected neuron was locally irradiated, repeatedly for 4 min, using a 488 nm laser in the spot indicated by a white circle (with a diameter ~1.7 μm). Images were recorded at 561 nm and using bright field optics. Following 20 min without activation, the 488 nm spot irradiation was repeated. Confocal images at various stages are shown. White arrows indicate the membrane protrusion area. (**f**) Kymograph along the line m-n at the activation site shows more ruffling and expansion activity during the illumination period. Kymograph along the line r-s shows that ruffling activity was suppressed on the opposite side of the neuron during the illumination period. Frame rate: 5 s/frame. Scale bars: 10 μm.

We tested whether opto-Dab1-FP would activate signaling and induce membrane protrusions in cortical neurons. We expressed opto-Dab1-mCh in primary embryonic cortical neurons by nucleofection, starved the cells, and illuminated with a blue light LED array. Western blotting showed that blue light induced phosphorylation of opto-Dab1-mCh, dependent on SFK activity (Supplementary Fig. S3). We then tested whether downstream effectors are activated. Neurons were nucleofected with opto-Dab1-GFP together with Akt-PH-mCh, Crk-SH2-mCh, or RalGDS-RBD-mCh (a probe for active Rap1^30^), then imaged using TIRF microscopy. Global 488 nm irradiation induced membrane translocation of opto-Dab1-GFP and each of the probes (Fig. 5a–f). This suggests that opto-Dab1 activation stimulates PI3K, Crk, and Rap1 in neurons. Next, we asked whether we could induce membrane protrusion by locally activating opto-Dab1 in neurons. As shown in Fig. 6a and b, local irradiation led to pronounced local membrane ruffling and expansion (Movie S5–6). Membrane ruffling and protrusion ceased when illumination was stopped, and restarted when illumination was restarted. As in COS7 cells, the membrane retracted on the opposite side of the cell (Fig. 6f, Movie S7). This may partly be due to competition for PI3K and Crk proteins at the activation site and partly due to membrane tension^31^,^32^. Together, the above results demonstrated that opto-Dab1 can induce cell motility with a high spatial resolution.

## Discussion

Reelin is thought to control neuronal migration by stimulating Dab1 phosphorylation and activating key pathways that regulate cytoskeletal dynamics, including PI3K, Rac1 and Rap1^6^,^7^,^8^,^9^,^10^,^11^,^12^,^13^,^33^. Dab1 is necessary for most Reelin signaling *in vivo*, but may not be sufficient. In principle, other Reelin-dependent but Dab1-independent pathways may contribute. Indeed, Reelin activation of Erk is only partially dependent on Dab1^34^. Also, Dab1 may also mediate signaling from other external stimuli, including amyloid precursor protein (APP) regulation of neuron migration and activated protein C stimulation of pro-coagulant activity of monocytes^35^,^36^. Here we showed that optogenetic activation of Dab1 is sufficient to recruit Crk to the membrane and stimulate Src, PI3K, Akt and Rap1. Local illumination of opto-Dab1 increased Akt recruitment up to 10 μm distant, and increased membrane ruffling and protrusion nearby. On the opposite side of the cell, ruffling was inhibited and membrane retraction was observed. Repeated activation of opto-Dab1 induced cell migration, similar to that observed with optically activated Rac1 and PLEKHG3^37^,^38^. These results suggest that local activation of Dab1 can induce polarization of intracellular signals and morphological asymmetry. Even though the developing brain contains many structural and environmental cues that help orient neuron migration^39^, these may not be needed if Dab1 is activated locally. Moreover, potential Dab1-independent, Reelin-dependent pathways are dispensable, at least under the conditions studied here. Directional Reelin-induced protrusive activity may be important as neurons transition from multipolar to bipolar migration in the intermediate zone and for leading process extension and somal translocation during in the marginal zone^8^,^10^,^11^,^39^. In this way, Reelin may be an instructive signal for neuron migration, rather than acting permissively to allow cells to respond to other directional cues.

The optogenetic system has several advantages over other activation methods as a tool to identify cellular effects of activated Dab1. (i) The activation process is fast (several seconds) and light dose dependent. This is due to the fast clustering kinetics of Cry2olig and the high affinity of clustered opto-Dab1 for plasma membrane. (ii) Activation is reversible with a t_1/2_ of minutes. This allows measurement of inactivation rates. (iii) Light can be directed with high spatial resolution and without diffusion that occurs with chemical activators. Although opto-Dab1 is a cytosolic protein, the increased avidity of clustered opto-Dab1 may limit its diffusion from the original activation area. Any opto-Dab1 molecules that do diffuse away are likely to be rapidly inactivated by protein-tyrosine phosphatases. Downstream signaling molecules activated by opto-Dab1, may also spread beyond the initial site of activation, as seen for PIP_3_ in our experiments, but their range is also limited by negative feedback mechanisms.

While our studies have used cultured cells, opto-Dab1 can, in principle, be used in more complex systems, such as *in vivo* neuron migration assays, which would give more detailed information about the role of Reelin signaling pathway in neuron migration. Opto-Dab1 may also be useful to study Reelin-Dab1 signaling in synaptic plasticity^1^,^40^, in the development of non-neuronal tissues, such as mammary gland, small intestine and limbs, and in diseases including cancer^41^,^42^,^43^. These applications will require the development of techniques to express opto-Dab1 effectively in the target cell types and to direct light to the respective tissues.

## Methods

### Plasmids

CRY2olig-mCherry was a gift from Chandra Tucker (Addgene plasmid # 60032). Dab1 or Dab1^5F^ (ref. 25) and various fluorescent proteins (FP) were amplified and inserted into the CRY2olig-mCherry plasmid between the NheI and XhoI sites (Dab1) and XmaI and NotI sites (FP) to make Dab1/Dab1^5F^-Cry2olig-FP (opto-Dab1/Dab1^5F^-FP). We then moved a NheI to NotI fragment from Dab1/Dab1^5F^-Cry2olig-FP into the pCAG vector to get pCAG-opto-Dab1/Dab1^5F^-FP, which was used for transfection and nucleofection.

Akt-PH-GFP was a gift from Tamas Balla (Addgene plasmid # 51465). Akt-PH, Crk-SH2, and RalGDS-RBD were amplified and inserted into pCAG-mCherry vector to make pCAG-Akt-PH-mCherry, pCAG-Crk-SH2-mCherry, and pCAG-RalGDS-RBD-mCherry, respectively. mCherry-Lifeact-7 was a gift from Michael Davidson (Addgene plasmid # 54491).

### Cell culture and transfection and nucleofection

HEK293, COS7 and NIH3T3 cells were maintained in DMEM containing 10% FBS and penicillin/streptomycin (both at 100 units/ml) in a humidified incubator with 5% CO_2_. Primary cortical neurons were prepared from embryonic day 15 (E15) mouse and kept in Neurobasal medium with 2% B27^8^. The imaging buffer for HEK293, COS7 and NIH3T3 cells was phenol red-free DMEM (Thermo Fisher 21063029). The imaging buffer for cortical neurons was phenol red-free Neurobasal medium (Thermo Fisher 12348017) supplemented with 10 mM HEPES and 1% Glutamax (Thermo Fisher 35050061). Plasmid transfection of HEK293 and COS7 cells was done according to the Lipofectamine 2000 protocol (Thermo Fisher Scientific Inc., #11668019). Nucleofection of cortical neuron followed the Amaxa nucleofector protocol. Briefly, ~4 × 10^6^ cells were centrifuged at 800 g for 4 min and resuspended with 100 μl Mirus Ingenio electroporation solution and 3 μg DNA. The cell/DNA suspension was then transferred to a cuvette and subjected to nucleofection using Program O-05.

### Antibody and chemicals

The primary antibodies used are as follows. 4G10 hybridoma supernatant (Fred Hutchinson antibody resource), Phospho-Akt (Ser473) antibody (Cell Signaling Technology, Inc., #9271), Akt Antibody (Cell Signaling Technology, Inc., #9272), Phospho-Src Family (Tyr416) Antibody (Cell Signaling Technology, Inc., #2101), Src antibody (monoclonal 327^44^), GAPDH antibody (Santa Cruz Biotechnology, Inc., #sc-25778), anti-mCherry hybridoma supernatant (kindly provided by Ben Hoffstrom and Jihong Bai). All the secondary antibodies are from Jackson ImmunoResearch Inc. The coating proteins used are as follows: fibronectin (Sigma-Aldrich Co. LLC., #F1141), collagen (Sigma-Aldrich Co. LLC., #C5533), poly-L-lysine (Sigma-Aldrich Co. LLC., #P2636). Other chemicals used are as follows: wortmannin (Sigma-Aldrich Co. LLC., #W1628), PP2 (EMD Millipore Corporation., #529573).

### LED array

The LED array for illuminating cells for biochemistry was made according to the methods as described^45^,^46^. Briefly, 6 LED bulbs and the BuckPuck LED driver were connected to an Arduino Uno microcontroller. Then the microcontroller was programmed through the open-source Arduino Software. Typically, illumination was for 0.05 s every 10 s.

### Confocal imaging

Cells were seeded on the coated glass-bottom dishes (World Precision Instruments, #FD35-100) and the medium was replaced with balanced phenol red-free medium. Then the dish was mounted in a live cell chamber (Live Cell Instrument, FC-5N model) placed on the stage of Zeiss LSM780 microscopy. The images were captured with a 63 × 1.4 NA objective. For global activation, the mCherry signal and the GFP signal (or EYFP signal) were acquired in a line sequential scan mode with excitation laser line 561 nm at 1% power and 488 nm at 0.2% power, respectively. Opto-Dab1 was activated, and images were collected, every 5–10 s, as noted in the figures. For the dark environment recovery, only mCherry signal was acquired. For local activation, a small spot was chosen and 10 iterations of 488 nm laser with 0.2% power and 0.79 μs duration time was used for each activation cycle after each 561 nm laser scan to acquire the mCherry signal. (For the NIH3T3 cell migration experiment, only 2 iterations of 488 nm laser illumination were used for each activation cycle). When EYFP-tagged opto-Dab1 was used in local activation, the EYFP signal was recorded only before and after the activation process (Fig. 3). For HEK293 cells and cortical neurons, images were acquired with a 2.0 zoom.

### TIRF imaging

TIRF imaging was used to achieve the global activation and observation near the ventral surface of the cell. Briefly, the glass-bottom dish was mounted in a live cell chamber (Live Cell Instrument, FC-5N model) placed on the stage of Nikon-TIRF microscopy. The images were captured with a 100 × 1.4 NA objective. mCherry and GFP signals were acquired by a CCD sequentially with excitation laser line 561 nm and 488 nm, respectively. Opto-Dab1 was activated, and images were collected, at 5 s intervals.

### Image analysis and statistics

Redistribution of fluorescence between cytosol and plasma membrane (confocal images), intensity changes near the ventral membrane (TIRF images), and protrusion/retraction analysis were quantified using Image J (https://imagej.nih.gov/ij/). Quantification and statistical analysis were performed using Prism 6 (GraphPad Software).

## Additional Information

**How to cite this article**: Wang, L. and Cooper, J. A. Optogenetic control of the Dab1 signaling pathway. *Sci. Rep.* **7**, 43760; doi: 10.1038/srep43760 (2017).

**Publisher's note:** Springer Nature remains neutral with regard to jurisdictional claims in published maps and institutional affiliations.

## Supplementary Material

Supplementary Movie S1

Supplementary Movie S2

Supplementary Movie S3

Supplementary Movie S4

Supplementary Movie S5

Supplementary Movie S6

Supplementary Movie S7

Supplementary Information
