# Supplementary material for: Optogenetic control of the Dab1 signaling pathway
Source: Sci Rep. 2017 Mar 8;7:43760. doi: 10.1038/srep43760 (PMC5363252; doi:10.1038/srep43760)
Supplement: Supplementary Information [file srep43760-s8.pdf]

Supplementary material for
Optogenetic control of the Dab1 signaling pathway

Liang Wang and Jonathan A. Cooper *

Division of Basic Sciences, Fred Hutchinson Cancer Research Center, 1100 Fairview Ave H, Seattle, Washington 98109, USA.

*Corresponding author: jcooper@fhcrc.org

Supplementary Information

Supplementary Information (text, tables and images) should be combined and supplied as a single file, preferably in PDF format.

Supplementary Fig. S1. Western blot analysis of phosphorylation level of opto-Dab1-mCh or opto-Dab1^{5F}-mCh. HEK293 cells expressing of opto-Dab1-mCh or opto-Dab1^{5F}-mCh were starved in serum-free medium for 15 hours, then left in the dark environment or illuminated by a LED array for 30 min (on/off, 0.05 s/12 s). Cell lysates were analyzed by Western blotting, probing with 4G10 anti-pY and anti-mCherry monoclonal antibodies.

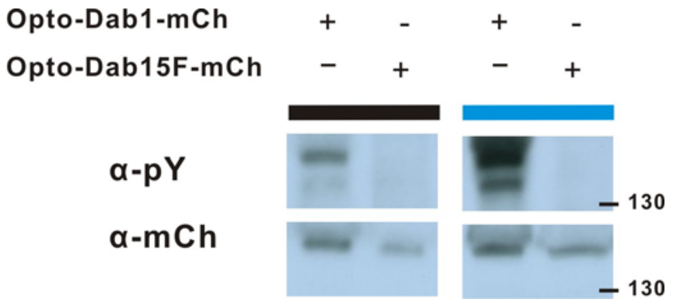

Supplementary Fig. S2. (A) Serum-starved COS7 cells expressing Akt-PH-mCh only were imaged before (0 s) and after 800 s of global blue light illumination (8 s/frame). (B) Reversible activation of PI3K in COS7 cells. TIRF imaging of PI3K activation during blue light irradiation of opto-Dab1-GFP. TIRP images were taken at the end of each activation/relaxation period. (C) Opto-Dab1^{5F}-FP mutant cannot recruit Crk-SH2-mCh probe. Cells expressing opto-Dab1^{5F}-EYFP and Crk-SH2-mCh were illuminated at 488 nm. (D) Protrusion can be induced by local irradiation at different sites in a single cell. Different spots (~ 6.6 μm diameter, white circles) were illuminated (488 nm, 0.2 Hz) on the edges of a serum-starved COS7 cell co-expressing opto-Dab1-EYFP and Akt-PH-mCh. Confocal images of the Akt-PH-mCh signal at the end of each light or dark period are shown. Scale bars: 10 μm.

Fig S2 (legend on previous page)

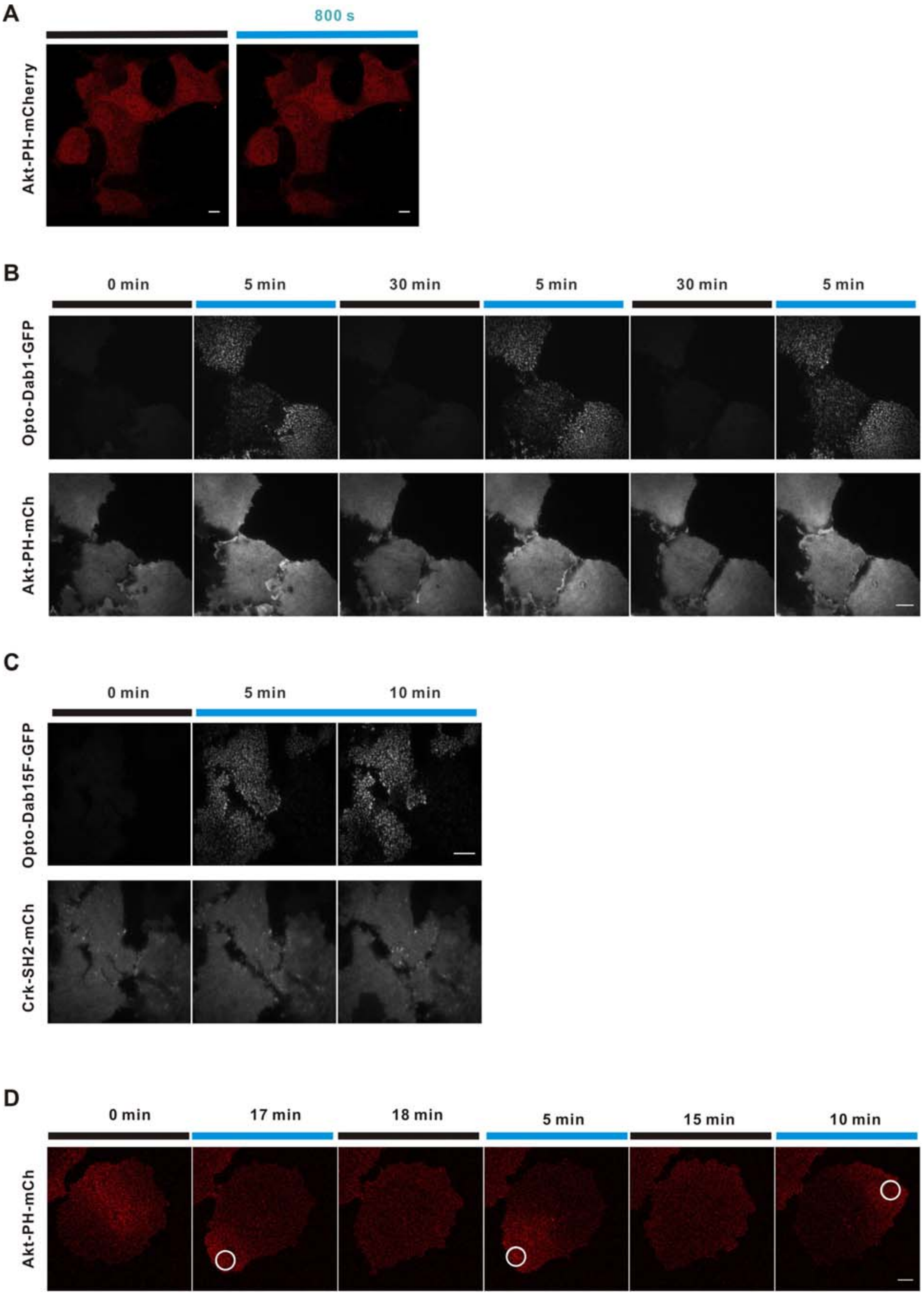

Supplementary Fig. S3. Phosphorylation of opto-Dab1-mCh induced by blue light in primary cortical neurons. Cortical neurons were prepared, nucleofected with opto-Dab1-mCh, and seeded on coverslips. Two days later, the neurons were starved in B27-free medium for 6 hours. Then the neurons were treated with 0.5 μM wortmannin or 10 μM PP2 or untreated and subjected to dark environment (in an incubator) or irradiation by a blue light LED array for 20 min (on/off; 0.05 s/10 s). Cell lysates were analyzed by Western blotting with anti-pY and anti-mCherry antibodies. The arrow indicates pY opto-Dab1-mCh.

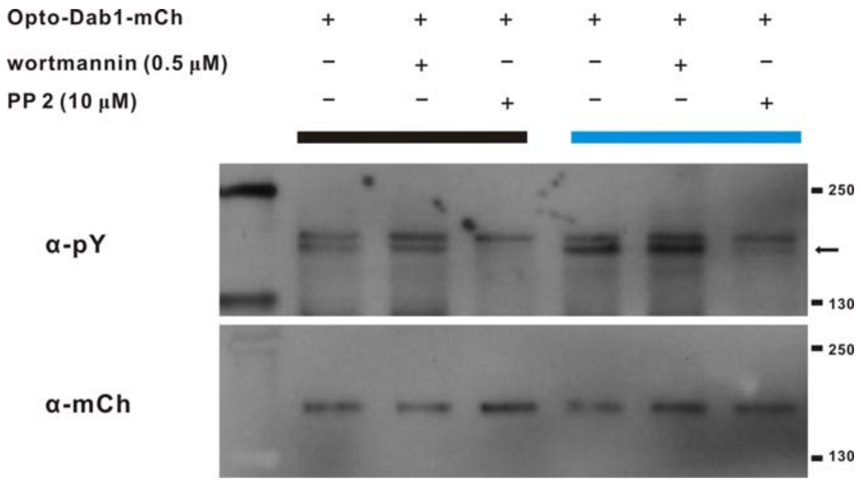

Supplementary movie S1 Protrusion induced by local activation of opto-Dab1-mCh near the edge of a COS7 cell.

Supplementary movie S2 Protrusion/Retraction induced by local activation/relaxation of opto-Dab1-mCh near the edge of a COS7 cell.

Supplementary movie S3 Local activation of opto-Dab1^{5F}-mCh near the edge of a COS7 cell induced opto-Dab1 translocation but no protrusion or ruffling.

Supplementary movie S4 Induction of NIH3T3 cell migration by repeated local activation. 01: cell expressing opto-Dab1-EYFP and Lifeact-mCh (which is shown in red fluorescence channel). 02: cell expressing opto-Dab1-mCh only.

Supplementary movie S5 Protrusion induced by local activation of opto-Dab1-mCh near a neuron cell body.

Supplementary movie S6 Protrusion induced by local activation of opto-Dab1-mCh at a neurite.

Supplementary movie S7 Activation and relaxation experiment in an early stage neuron expressing opto-Dab1-mCh.
